# Single-image dehazing method based on Rayleigh Scattering and adaptive color compensation

**DOI:** 10.1371/journal.pone.0315176

**Published:** 2025-03-20

**Authors:** Xin Guo, Qilong Sun, Jinghua Zhao, Mingchen Sun, Yiyang Qiao, Yingying Zhang, Yan Zhou

**Affiliations:** 1 School of Mathematics and Computer Science, Jilin Normal University, Siping, Jilin, China; 2 Personnel Division, Jilin Normal University, Siping, Jilin, China; 3 School of Computer Science and Technology, Jilin University, Changchun, Jilin, China; University of Electronic Science and Technology of China, CHINA

## Abstract

We propose a Rayleigh Scattering and adaptive color compensation method. It capitalizes on the brightness and color differentials between the regions where DCP has failed within images for effective regional segmentation. First, we added B-channel compensation to the atmospheric illumination, made a simple evaluation of the B channel through the atmospheric illumination of the R channel and the G channel. It repeatedly iterated to obtain and repaired the atmospheric illumination of the B channel, which eliminates the color dilution. Secondly, we obtained the dark channel image and the bright channel image, and jointly evaluated the failure point of the dark channel prior method to select the area with inaccurate transmission. This can select the areas which need re-estimate the transmission. This step improves the image quality of the area and repairs the image details. Finally, we validated the effectiveness and resilience of the proposed method through comprehensive experiments. It is conducted across diverse scenarios, involving the adjustment of various parameters.

## Introduction

Air pollution leads to an increase in suspended particles in the atmosphere, which results in reduced visibility. It creates the natural atmospheric phenomena known as fog and haze. Light rays undergo multiple interactions with airborne particles due to fog and haze, which leads to a decrease in light intensity. This impacts imaging devices and results in images and videos with reduced brightness, lower contrast and color distortion. The development of defogging techniques is crucial to mitigate these challenges in the image processing.

In recent years, image defogging methods are mainly divided into multi-image defogging methods [1] and single-image defogging methods. Multi-image defogging methods are limited to a defogging scenario that requires multi-angle images, which greatly restricts the development of this direction. In contrast to multi-image dehazing methods, which leverage multiple images of the same scene captured from different angles and under varying weather conditions, single-image dehazing lacks this abundance of data. Various types of hazy images exist, each with complex and unpredictable depth of field. Single-image dehazing methods have made significant advancements, and their main methods can be broadly categorized into three types: image dehazing methods based on image enhancement [2–4], image dehazing methods based on image fusion [5–7], and image dehazing methods based on physical models [8–11].

The last category of dehazing methods is based on physical models. One noteworthy contribution to the field is the Dark Channel Prior (DCP) [12] introduced by He et al. DCP is grounded in the atmospheric scattering model and stems from extensive observations of outdoor images. The theory notes a tendency toward zero values in the three color channels of pixels within non-bright regions of clear outdoor images. And researchers extended the DCP theory, introducing the Bright Channel Prior (BCP) [13] theory. Their investigations confirmed that fog concentration is directly linked to the difference in brightness and saturation. This led to the amalgamation of BCP and DCP, resulting in the proposal of various improved methods.

Eﬃcient image defogging methods are still a topic of research in the computer vision. As described, compared with other methods, the image dehazing method based on the physical model relies on a prior theory. This determines that accurate transmission and atmospheric illuminations are key to obtaining clear images. We propose a single image defogging method based on Rayleigh Scattering and adaptive color compensation(RSA) for repairing transmission and atmospheric illuminations. The RSA takes a foggy image as an input, and adaptively adjusts. On the one hand, the atmospheric illumination and transmission by using the Histogram Correlation Coeﬃcient(HCC) objective parameter as an adaptive operator improves the quality of the restored image. This process fixes the image chromatic aberration problem and makes the dehazed image as close to the original image as possible. The method reduces the problem of excessive color deviation and corrects the color difference of image objects caused by the dehazing method. It lays a solid foundation for the next step to estimate the accurate transmission. On the other hand, we use DCP and BCP to get the reference image to filter out areas where the transmission estimation is inaccurate and improve the image’s ability to express details.

Our main contributions can be summarized as follows.

- We propose a new adaptive adjustment framework based on the DCP theory, which adaptively adjusts the atmospheric illuminations for Rayleigh compensation, addressing the color deviation issues in dehazed images.
- We research an in-depth study of the DCP theory, extract areas where the DCP fails, and further optimize image quality.
- We select multiple hazy images, and highlight the effectiveness of our method based on six objective parameters, focusing on the adaptation to atmospheric illuminations.

## Related work

To depict the image formation process in foggy conditions, the atmospheric scattering model serves as a fundamental reference. McCartney et al. [14] introduced a prototype atmospheric scattering model in 1976, and it is later refined by Narasimhan and Nayar [15,16]. Most image dehazing methods based on physical models are proposed on the basis of the atmospheric scattering model. Since the equation has two unknown quantities, it is great uncertain.

Tan et al. [17] believed that clear images have higher clarity and contrast than hazy images, and the changes in atmospheric illuminations are softer. They used white balance to improve hazy images, and used the Maldives Random Fields [18] to estimate the atmospheric illuminations. However, it maximizes the contrast of the image in the process of estimating the atmospheric illuminations, distorting the color, and due to the block operation, which causes a halo phenomenon.

He et al. [12] made a significant contribution by introducing the concept of the dark channel image(DCI) in their research. Their meticulous examination of numerous clear outdoor images unveiled a compelling discovery. In the majority of pixels within these images, one of the three RGB channels consistently displayed remarkably low pixel values, often approaching 0. Yan et al. [13] provided a comprehensive summary of the DCP theory. Their analysis revealed a consistent maximum value in the three channels of a clear image, closely approximating 255. Building upon this observation, they proposed the BCP theory, marking the beginning of physically-based image defogging methods. Chu et al. [19] introduced DBCP-III as an assessment tool for image quality post-defogging, which leverages the DCP and BCP defogging effects. Further strides in haze removal emerged, including a versatile method effectively removes fog from both daytime outdoor images and nighttime foggy scenes. Li et al. [20] addressed foggy images through image segmentation techniques, estimating atmospheric illuminations and transmission for bright regions, deploying enhanced BCP and DCP methodologies. These estimates are further refined using Gradient Domain Guided Filtering. While these innovations represent significant progress, they do exhibit certain limitations, particularly in restoring brightness in specific image regions, leading to potentially unsatisfactory defogging outcomes. Although DCP has high processing eﬃciency, the three point values may not tend to zero when selecting pixels in bright areas. It results in DCP being unable to select dark channel images in this area, causing the method to fail. In addition, due to the failure of DCI, the method also has deviations when it selects atmospheric illumination, which affects the estimation of transmission in turn. Most of the methods improved based on DCP can’t get rid of the constraints of this theory, which results in high saturation and poor brightness of the repaired image, halo phenomenon in bright areas, and introduction of noise.

Zhu et al. [21] conducted a thorough examination of numerous images. Their findings indicated that atmospheric illumination was enhanced when an image was covered by fog. This enhancement made it challenging to discern the original color of objects. They observed that as the fog concentration in the image increased, this phenomenon became more pronounced. Through detailed analysis of areas with varying fog concentrations, they discovered that high-concentration areas exhibit characteristics of high brightness and low saturation. The conclusion drawn from this observation is that the concentration of fog is proportional to the difference between brightness and saturation. This theory has undergone widespread refinement and application [22,23], which showcases its adaptability and relevance across various scenarios. The methods based on color attenuation prior obtain the image transmission based on the difference between the brightness and saturation of the outdoor image and the fog concentration. It has a good defogging effect on hazy images with clear backgrounds, but the images processed by this method are prone to additional noise points, and the enhancement effect around the fog group is poor. It is worth noting that Sahu et al. [24] proposed a unique based on color end-to-end dehazing network for restoring clear images from their counterparts without using atmospheric scattering models.

However, these methods often deviate from reality in estimating the the atmospheric illuminations and transmission process. The gap between actual value and estimated value leads to problems such as color deviation and poor brightness in the images. For example, the DCP often includes high pixel values in non-sky regions in the estimation of the atmospheric illuminations, which causes a deviation between the estimated and actual values. The image’s transmission rate is calculated based on the atmospheric illuminations. It leads to corresponding deviations and this ultimately results in a globally lower brightness of the dehazed image, with halo phenomena in the sky, significantly reducing the overall image quality. To address these issues, improve image quality, and fix color deviation, we adapt the atmospheric illuminations based on Rayleigh Scattering, perform color compensation, and optimize color deviation issues. By combining DCI and BCI, we identify the areas where DCP fails, repair the transmission rate in those regions, enhance image quality, and improve the unsatisfactory restoration in the sky region.

## Methods

**Fig 1:**
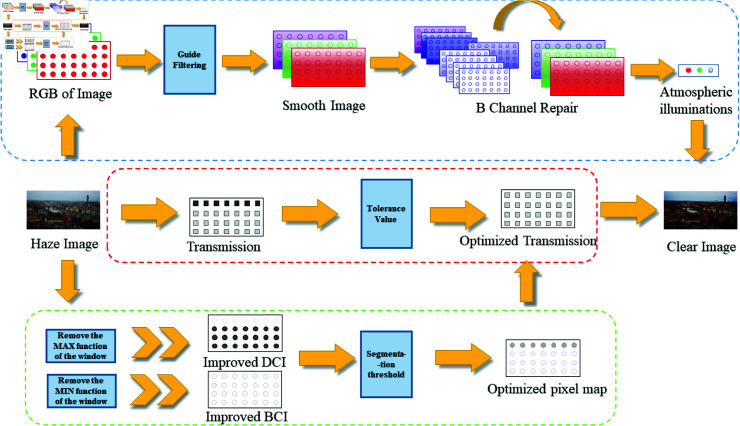
Method flow image. This image depicts the actual flow of the method.

In this part, we present a single-image dehazing method based on Rayleigh Scattering and adaptive color compensation(RSA). The RSA adaptively compensates the atmospheric illumination of the B-channel with the HCC operator and derives the reference image using the BCP and DCP. The method identifies the regions where DCP theory fails in the image through adaptive segmentation threshold adjustments of the HCC operator. Moreover, it optimizes the transmission of these regions using a tolerance parameter and finally computes the clear image using the atmospheric scattering. Compared with other methods, ours has made great changes in image color restoration. By continuously iterating and selecting the optimal solution for atmospheric illuminations, it also reduces the error in transmission and further optimizes the image quality. Since most of the current physical model-based dehazing methods are based on the DCP, and they cannot get rid of the limitation of regional failure in extremely bright areas. We combine DCI with the bright channel image(BCI), adjust the pixels where DCP fails, and optimize the transmission of the area. The process is shown in the Fig 1.

### Atmospheric illumination

We employ a smoothing function on the original image by applying the guided filter [25]. The guide filter is a linear filter that maintains the gradient value of the image while ensuring that the smoothed image retains the characteristics of both the original and smoothed images. This approach guarantees that the quality of the smoothed image, denoted as *S*, closely resembles that of the filtered image. It also reduces the relative differences in pixel values between the images and eliminates the influence of speckle noise on the selection of atmospheric illuminations. The process is shown in Eq (1).


$$\begin{array}{ccc} S=GUIDEFILTER(I,q,r,\delta ), & & \end{array}$$ (1)

where *S* represents the smoothed image, *GUIDEFILTER*() denotes guided filtering, *I* represents the haze image, *q* stands for the guide image, *r* is the window size, and *δ* is the regularization parameter. To obtain the atmospheric illumination *A* from the smoothed image [26], the first 0.1*%* of pixel points are selected. However, it’s essential to note that, according to Rayleigh Scattering theory, blue light in the imaging spectrum is more susceptible to dispersion effects in foggy conditions. Deviations in the atmospheric illumination can significantly impact image color, brightness, and other quality attributes.

In any image, there exists an atmospheric illumination threshold, where the objective parameter that describes image color, brightness, and other qualities reaches an extreme value. When the estimated atmospheric illumination falls below or exceeds this threshold, the objective parameter deviates from its extreme value. The HCC [27] is an objective parameter used to gauge image similarity, with higher values indicating a higher degree of color similarity between two images.

To enhance the accuracy of the estimated atmospheric illumination, the B-color channel should undergo adaptive compensation. The HCC objective parameter is employed to adjust the B-color channel continuously, altering its value based on HCC as a criterion. This process aims to select the B-color channel value that results in the highest HCC objective parameter. The following Eq (2) demonstrates this process.


$$\begin{array}{ccc} A\{\begin{array}{ccc} & A_{r}=A_{R} & \\ & A_{g}=A_{G}\\ & A_{b}=APT(A_{B}) \end{array}, & & \end{array}$$ (2)

where *A* represents the adjusted atmospheric illumination, $A_{R}$, $A_{G}$, and $A_{B}$ represent the atmospheric illuminations of the three color channels of $A_{i}$ before adjustment, and $A_{r}$, $A_{g}$, and $A_{b}$ represent the atmospheric illuminations of the three color channels of $A_{i}$ after optimization. *APT*() is the adaptive tuning function that iteratively replaces the old value with the new one until the HCC reaches its extreme.

### Optimization of transmission using the reference image segmentation method

There will always be a channel with a relatively low or high pixel value for most pixels in the three channels of clear outdoor images, approaching 0 or 255 respectively, through the analysis of the DCP theory and the BCP theory. However, some pixels have high values in all three channels, which causes the DCP theory to fail. It results in inaccurate estimates of the transmission rate through this theory. To accurately find the regions that need optimization for transmission rates, RSA selects the regions optimized for brightness in the DCI and BCI.

**Fig 2:**
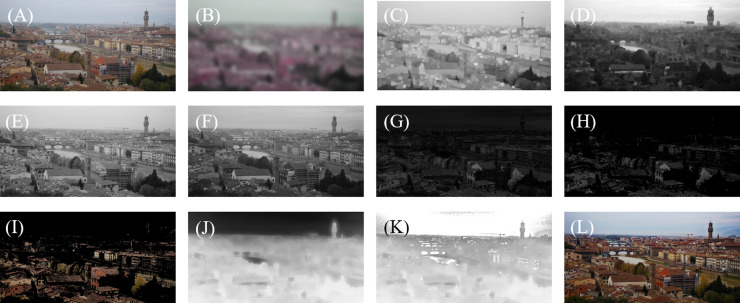
Method image. (A) is the image that need to be defogged. (B) is the guide image for calculating atmospheric illuminations. (C) and (D) are conventional channel images. (E) and (F) are improved channel images. (G) is the image used to find failed areas(H) and (I) select the pixels to be optimized from (J) based on the segmentation threshold. (J), (K) are the transmission before and after optimization respectively. (L) is the clear image recovered.

However, due to the inclusion of a window during the selection of channel images, the DCI and the BCI become blocky. The window makes it challenging to accurately select the precise regions needed improving. As shown in Fig 2C and 2D represent conventional channel images, the BCI and DCI have been blurred by the window, which causes the image details to become blocky. It is diﬃcult to judge whether the image details need optimization.

Therefore, when selecting the DCI and BCI, we removed the selection window to allow the selection of improved channel images. As shown in Fig 2E and 2F, these are the improved channel images. By observing, we can see that the improved BCI and DCI have undergone significant changes in detail. This reveals the contours of objects and preserving details well. After subtracting the improved BCI from the DCI, the resulting image is named the reference image *R*(*x,y*), as shown in Fig 2G.

Most pixel values in the BCI are close to 255, while they are close to 0 in the DCI. However, the DCP does not hold in areas that need optimization, and the pixel values are relatively large. Therefore, the areas with very small values correspond to the regions that need optimization for transmission rates of the haze image in the reference image *R*(*x,y*). To complete this step, a segmentation threshold *φ* established. The threshold is adaptively adjusted using HCC objective parameter to get Fig 2H and 2I.

According to Fig 2H and 2I, we obtained the set of pixels that require transmission optimization due to the failure of the DCP. We optimized them through the tolerance value and obtained Fig 2K. By comparing Fig 2J and 2K, we can find that the transmission in areas such as the sky and under the bridge is effectively optimized. After obtaining the above parameters, we can calculate the clear image *J*(*x,y*) through Eq (3).


$$\begin{array}{ccc} J(x,y)\{\begin{array}{ccc} & \frac{I(x,y)-A}{\frac{K}{I(x,y)}\cdot \max(t(x,y),t_{0})}+A\text{,}R(m,n)\geq \varphi & \\ & \frac{I(x,y)-A}{\max(t(x,y),t_{0})}+A\text{,}R(x,y)<\varphi \end{array}, & & \end{array}$$ (3)

where *I*(*x,y*) represents the hazy image, *J*(*x,y*) represents the clear image, *K* is the tolerance value [28], *R*(*x,y*) is the reference image, *A* denotes the atmospheric illumination, *φ* is a segmentation threshold, and *t*(*x,y*) stands for transmission. A constant $t_{0}$ is introduced to prevent the transmission from being zero, which would render the formula meaningless. Additionally, *x* and *y* represent the horizontal and vertical coordinates of the pixel points, respectively.

## Experiments and result

In this section, we aim to demonstrate the validity of the experiment by assessing both subjective visual effects and objective parameters. The images in our method are all from free and open source databases provided by method [29], method [30], method [31], and method [32]. The dataset contains haze free images, synthetic distance maps and corresponding simulated haze images [31]. The goal is to illustrate the method’s effectiveness and versatility. We selected an image that encompasses a town and natural landscape for this purpose. Furthermore, we started a comparative analysis by evaluating objective parameters obtained from the processed image.

### Experimental environment and objective parameters

Our experiments are proposed on an ordinary PC computer with a 64-bit operating system. The detailed configuration is that the processor is Intel(R) Core(TM) i7-6700 CPU @ 3.40GHz 3.40GHz, the system memory is 8GB, the environment is Matlab2016. In order to further illustrate the effectiveness of RSA, we evaluate objectively from six parameters: SSIM (Structural SIMilarity), UQI (Universal Quality Index), PSNR (Peak Signal-To-Noise Ratio), AVE (Average), HCC and MS-SSIM illustrate.

SSIM objective parameters evaluate the quality of the restored image from three levels: brightness, contrast and image structure of the image. The brightness *L*(*x,y*), contrast *C*(*x,y*) and image structure *S*(*x,y*) of expression are Eqs (4)–(6).


$$\begin{array}{ll} & L(x,y)=\frac{2\mu_{I}\mu_{J}+C_{1}}{{U^{2}}_{J}+{U^{2}}_{\text{I}}+C_{1}},\; \end{array}$$ (4)

$$\begin{array}{ll} & C(x,y)=\frac{2\sigma_{I}\sigma_{J}+C_{2}}{{\sigma^{2}}_{J}+{\sigma^{2}}_{\text{I}}+C_{2}},\; \end{array}$$ (5)

$$\begin{array}{ll} & S(x,y)=\frac{\sigma_{IJ}+C_{3}}{\sigma_{I}\sigma_{J}+C_{3}},\; \end{array}$$ (6)

After obtaining the above parameters, the SSIM can be shown:


$$\begin{array}{ll} & \{\begin{array}{ccc} & C_{1}={\left(0.01T_{t}\right)}^{2} & \\ & C_{2}={\left(0.03T_{t}\right)}^{2}\\ & C_{3}=C_{2}∕2 \end{array},\; \end{array}$$ (7)

$$\begin{array}{ll} & \text{SSIM}(m,n)=L(m,n)*C(m,n)*S(m,n),\; \end{array}$$ (8)

where *I* and *J* are the hazy image and clear image respectively. $\mu_{I}$ and $\mu_{J}$ are the average of the two images, and $\sigma_{I}$, $\sigma_{J}$ are the variances of the two images. The $\sigma_{IJ}$ is the covariance of *I* and *J* of the image, and $T_{t}$ is the range of image pixel values. The *x*, *y* are the horizontal and vertical coordinates of the pixel respectively.

The expressions of PSNR are as shown in Eqs (9) and (10):


$$\begin{array}{ll} & \text{MSE}=\frac{1}{M*N}\overset{M}{\underset{x=1}{\sum }}\overset{N}{\underset{y=1}{\sum }}{\left(I(x,y)-J(x,y)\right)}^{2},\; \end{array}$$ (9)

PSNR=1M∗N10log ⁡ 10(max ⁡ I2MSE), (10)

where MSE (Mean Square Error) represents the mean square error of the hazy image *I* and the clear image *J*, *M* and *N* are the image sizes, and *x*, *y* are the horizontal and vertical coordinates of the image. The max ⁡ I is generally taken as 255. The expression of UQI is shown in Eq (11), assuming that the size of the image is *M* ∗  *N*, *x* and *y* are the horizontal and vertical coordinates of the image:


$$\begin{array}{ccc} \text{UQI}=\frac{4\sigma_{xy}\bar{xy}}{\left({\sigma^{2}}_{x}+{\sigma^{2}}_{y})[{\left(\bar{x}\right)}^{2}+{\left(\bar{y}\right)}^{2}\right]}, & & \end{array}$$ (11)

$\bar{x}$ and $\bar{y}$ are the average of *x* and *y*, which can be expressed as $\bar{x}=\frac{1}{M}\overset{M}{\underset{i=1}{\sum }}x_{i}$ and $\bar{y}=\frac{1}{N}\overset{N}{\underset{j=1}{\sum }}y_{j}$. $\sigma_{x}$ and $\sigma_{y}$ are the variance of the image in the *x* and *y* directions, $\sigma_{IJ}$ is the covariance of the image. They can be expressed as ${\sigma^{2}}_{x}=\frac{1}{M-1}\overset{M}{\underset{i=1}{\sum }}{\left(x_{i}-\bar{x}\right)}^{2}$ , ${\sigma^{2}}_{y}=\frac{1}{N-1}\overset{N}{\underset{j=1}{\sum }}{\left(y_{j}-\bar{y}\right)}^{2}$, ${\sigma_{x}^{2}}_{y}=[\frac{1}{M-1}\overset{M}{\underset{i=1}{\sum }}{\left(x_{i}-\bar{x}\right)}^{2}]\cdot [\frac{1}{N-1}\overset{N}{\underset{j=1}{\sum }}{\left(y_{j}-\bar{y}\right)}^{2}]$. Here *i* and *j* respectively play the role of counting. In order to better describe the characteristics of the three factors of comprehensive correlation loss, brightness distortion and contrast distortion of UQI parameters, UQI can be rewritten as Eq (12):


$$\begin{array}{ccc} \text{UQI}=\frac{\sigma_{xy}}{\sigma_{x}\sigma_{y}}\cdot \frac{2\bar{xy}}{{\left(\bar{x}\right)}^{2}+{\left(\bar{y}\right)}^{2}}\cdot \frac{2\sigma_{x}\sigma_{y}}{{\sigma_{x}}^{2}+{\sigma_{y}}^{2}}, & & \end{array}$$ (12)

AVE represents the average of image pixel values. In the same image, the images that are restored by different defogging methods contain different image information. Its internal brightness, color saturation and other image elements will interfere with the level of pixel values. The lower the pixel value, the lower its recovery quality, and its expression can be expressed as Eq (13).


$$\begin{array}{ccc} \text{AVE}=\frac{1}{M*N}\overset{M}{\underset{x=1}{\sum }}\overset{N}{\underset{y=1}{\sum }}E(x,y), & & \end{array}$$ (13)

where *x*, *y* are the horizontal and vertical coordinates of the image pixel, *M*, *N* are the length and width dimensions of the image, and *E*(*x*, *y*) is the pixel value of the pixel.

The HCC objective evaluation parameter is to compare the histograms of two images and determine the similarity of the two histograms through the correlation coeﬃcient of the histogram distribution. The higher the value, the higher the matching degree, which means the lower the image distortion rate and chromatic aberration.

The MS-SSIM is an algorithm used to compare the similarity between two images [33]. It is designed based on the perception principle of the human visual system on images and can effectively reflect the structural similarity of images [34,35]. The algorithm can be described as Eq (14)


$$\begin{array}{ccc} \text{MS-SSIM}={\left[L_{Z}(x,y)\right]}^{\sigma_{Z}}\cdot \overset{Z}{\underset{j=1}{\prod }}{\left[C_{j}(x,y)\right]}^{\beta_{j}}{\left[S_{j}(x,y)\right]}^{\gamma_{j}} & & \end{array}$$ (14)

Among them, $\left[L_{Z}(x,y)\right]$, *C*_*j*_(*x*, *y*) and $S_{j}(x,y)$ have the same meaning as the parameter SSIM in Eqs (5)–(7), Z is the three dimensions of the image, $\sigma_{Z}$, $\beta_{j}$ and $\gamma_{j}$ are used to adjust the relative importance of different components.

### Adaptive selection of atmospheric illumination

To validate the effectiveness of adaptive atmospheric illumination, we select two images from separate experiments and manually adjusted the atmospheric illuminations in the B channel. Subsequently, we compare these adjustments with the HCC objective parameters. Upon inspecting the images, it is easily observed that the image takes on a bluish overall hue, when the atmospheric illumination of the B channel is too small. Although the sky may see some improvement, the ordinary areas exhibit a noticeable blue bias. Conversely, when the atmospheric illumination in the B-color channel is excessively large, the non-bright areas of the image experience significant enhancement. The bright sky area may appear yellowish.

Upon evaluating the images and corresponding HCC objective parameters, we found that the B- channel atmospheric illumination tends to align with the average value of the R and G color channels, with a deviation of approximately +0.04. This leads to yellowing of the image and color distortion. These deviations generally manifest as quadratic functions and typically occur within a range of  ± 0.04 around the average values of the R and G color channels’ atmospheric illuminations. In the RSA, the atmospheric illumination is selected through adaptive optimization consistently yielded images with objective parameters that reached extreme values, confirming the eﬃcacy of ours.

**Fig 3:**
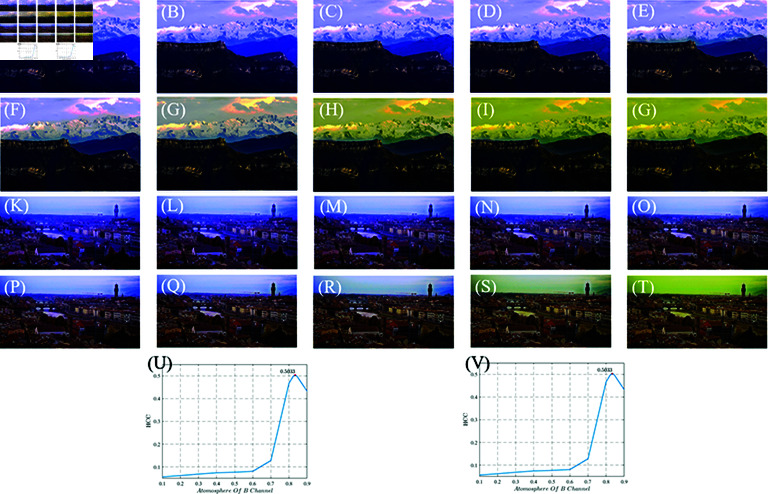
Atmospheric illumination variation image and their HCC-atmosphere of B channel line chart. (A)–(J) shows the Snow Mountain image $A_{b}$ gradually increasing, (K)–(T) shows the City image $A_{b}$ gradually increasing, (U) is the HCC-Atmosphere of B-color channel Line Chart of Mountain, (V) is the HCC-Atmosphere of B-color channel Line Chart of Mountain.

According to Fig 3, it is evident that as the atmospheric illumination in the B-color channel increases, the image gradually turns yellow. When the atmospheric illuminations of the Snow mountain image reaches 0.8, the image exhibits a noticeable deviation in color, and for the Town Image. And when the atmospheric illumination reaches 0.9, the image takes on a yellowish hue. From this, we can preliminarily infer that the atmospheric illuminations for the two images are less than 0.8 and 0.9, respectively. Furthermore, in the Snow mountain Image, the atmospheric illumination is below 0.6, and in the Town image, the atmospheric illumination is 0.8, both resulting in a distinct bluish tint. We roughly depict a line graph illustrating the variation of the HCC with changes in the atmospheric illumination in the B-color channel. And the HCC reaches extreme values at the same time. It aligns with the speculated range from subjective observation.

### Superiority test

**Fig 4:**
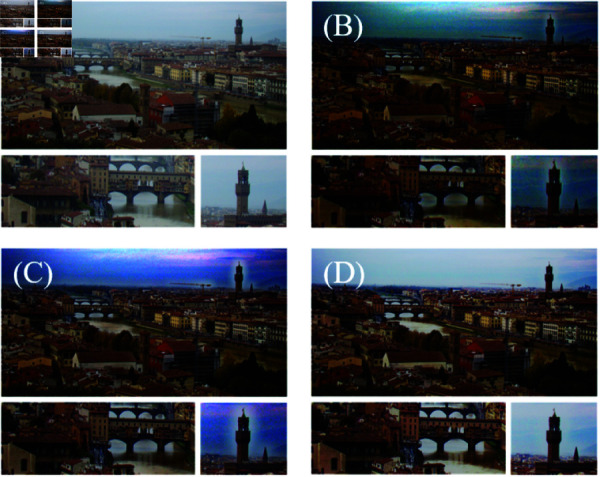
Experimental comparison chart. (A) The foggy image of the image. (B) The dehazed image after dark channel image processing. (C) An experimental dehazing image for replacing the algorithm in this paper with DCP transmission, which is Test Experiment 1. (D) RSA.

To evaluate the effectiveness of RSA, Test Experiment 1 is designed. The section keeps all other variables constant and replaces the improved transmission suggested in our method with the transmission proposed by DCP before performing defogging, named Test Experiment 1. Combine the hazy image, DCP processed image, Test Experiment 1 processed image, and the image processed thought ours to form Fig 4. Moreover compare it using the six objective parameters of PSNR, SSIM, AVE, UQI, HHC and MS-SSIM as shown in Table 1.

**Table 1:** Objective parameters of experimental comparison chart.

Objective parameters	DCP [12]	Test Experiment 1	RSA
PSNR	63.3504	64.6734	69.1043
SSIM	0.7513	0.8480	0.9902
AVE	0.2761	0.3301	0.3863
UQI	0.6624	0.8141	0.8702
HCC	− 0 . 0471	0.2560	0.5680

Upon Fig 4B and 4C, we can draw the following points.

- The brightness of Fig 4C has been improved. Its color saturation is closer to the original image.
- It is evident that the image details have improved in Test Experiment 1 upon closer inspection of the area near the bridge in both images, including the window details.
- When enlarging the tower part of the images, the details of the tower part of the image processed by Test Experiment 1 are clearer than the image repaired by DCP, and the color is more natural.
- Compare the objective parameters in Table 1. The parameters in Experiment 1 are higher than those in DCP.

These comparison demonstrates the effectiveness and robustness of the modified atmospheric illumination proposed in RSA. The analysis above further proves that plays a key role in repairing image color and improving image brightness to a certain extent in our RSA. However, there are still some problems in the processed images in Test Experiment 1.

- The color saturation of the repaired image is still too high. This seriously interferes with the expression of information in image details. The overall image is unnatural.
- There is still halo phenomenon in the sky of the image. When observing the river, it is not diﬃcult to find that noise is introduced.
- A white halo phenomenon appears around the tower.

The root cause of the above problems is that the transmission is not accurate enough. Due to an error in image transmission, the dehazing process is not fully effective. It results in a slightly foggy image with lighter colors. The outline of the tower clock in the tower is affected by inaccurate transmission of DCP and Test Experiment 1, which results in a white aperture. Our RSA has successfully corrected these issues. Examining Table 1, it is evident that several objective parameters have improved significantly. However, despite these factors, the AVE value of the image processed by ours is still higher than that of Test Experiment 1. This demonstrates the accuracy of the transmission estimated and further validates its effectiveness.

We previously introduced six objective parameters. Table 1 shows that the methods proposed have higher objective parameter measurements. The reason for this situation may be that some areas have a black outer frame in the image of Test Experiment 1. It indicates that the atmospheric illumination selected by the methods deviates from the actual value. This causes the estimated transmission to also deviate, resulting in errors in depth of field estimation, inaccurate color restoration, loss of details, and deviations from the original image. The loss of details causes a decrease in the objective parameters of PSNR, SSIM, UQI and MS-SSIM. Halos appear in the sky and a large amount of noise is introduced due to the inaccuracy of transmission, greatly affecting the evaluation of parameters. As a result, We can find that the improved transmission effectively improves the quality of the image.

**Fig 5:**
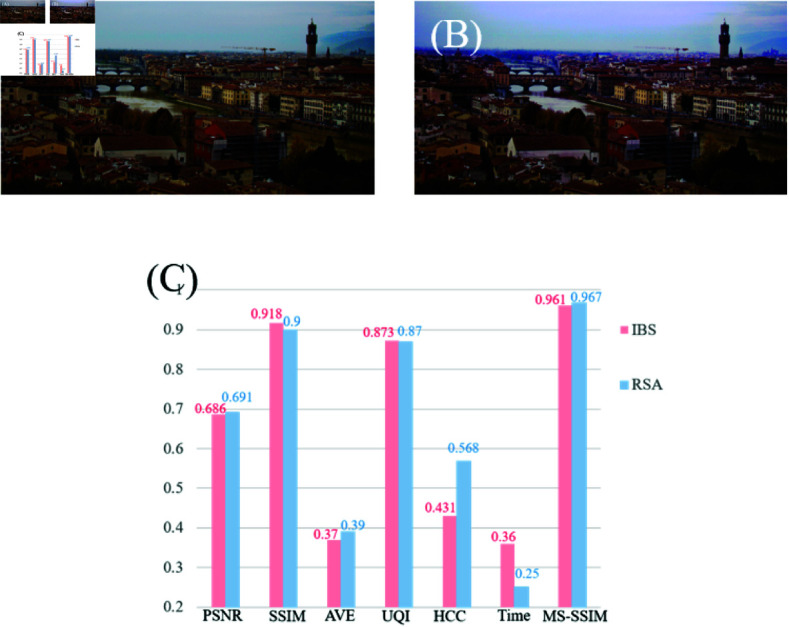
Our work comparison. (A) is IBS [36]. (B) is RSA. (C) is image objective parameter histogram.

Previously, we proposed dark channel image dehazing based on the improved binary image segmentation (IBS) [36]. In this work, we focus on improving the DCP method through a collection of methods to improve image quality. However, as explained above, the image repaired by the IBS will produce obvious boundaries at the edge of the image segmentation. This causes the observer to feel uncomfortable when observing the image, and the salt and pepper noise is scattered at the junction of the bright area and the ordinary area. Now, our work focuses on improving the DCP method itself, by finding and improving the DCP failure part, and repairing the transmittance of this part to further improve the image. Fig 5 is an image produced by the repaired images of the two methods we proposed and their corresponding objective parameters. For the convenience of observation, we normalized the data. We can see that this work has well avoided the noise problem at the boundary and further improved the image. By observing the bar graph, we can find that although the SSIM and UQI of IBS are slightly higher than RSA, the PSNR, AVE, HCC, and MS-SSIM are all lower than RSA. RSA is proposed to fix the color difference problem of the image and better ensure the authenticity and reliability of the image color. The improvement of the objective parameter HCC is enough to illustrate the effectiveness of RSA. In addition, RSA has also made corresponding improvements in algorithm operation eﬃciency.

### Qualitative results on real-world images

**Fig 6:**
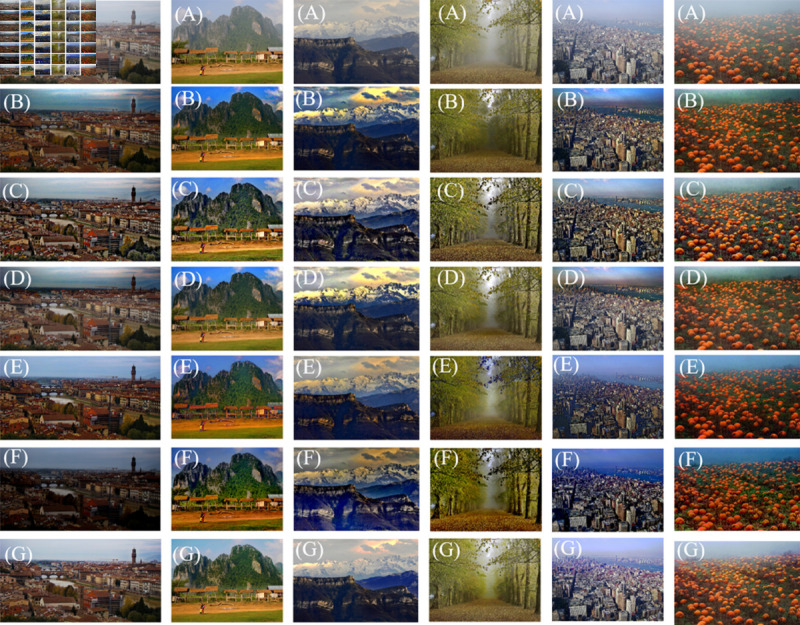
Qualitative comparison of different methods on real-world images. (A) are foggy images, (B) are images processed by DCP [12]. (C) are images optimized by MAMF [30]. (D) are images processed by TDD [37], (E) are images processed by PDE [38], and (F) are images processed by JCE [39]. (G) are RSA.

In defogging methods, improving the fog processing effect in the sky region has often proven challenging. This is attributed to the presence of bright areas or noise in the foggy images, which affects the accuracy of atmospheric illumination selection. Furthermore, the sky areas share visual characteristics with fog, which makes transmission estimation less precise. From the Fig 6, it is clear the follow.

- Due to the introduction of DCI, the DCP has an inaccurate evaluation of transmission in the process of image processing. It overestimates the transmission, causing the dehazed image to have too large depth of field and loss of details, and cannot handle areas with too many bright areas well. In addition, when there are too many bright areas, the atmospheric illumination is mistakenly selected to the bright area. It makes the atmospheric illuminations is larger than actual value. Because of the atmospheric illuminations’ issues, the transmission is further deviated. The DCP processed image exhibits a noticeable halo effect in the sky and overall reduced brightness, accompanied by excessive saturation. Although DCP has many shortcomings in image processing, it fundamentally provides a new idea for the traditional dehazed image method and has extremely fast processing.
- The MAMF input image is decomposed into intensity and Laplacian modules, which are enhanced at the pixel and gradient levels respectively. Since the detail layer guarantees the gradient information, the output image can produce results that guarantee details even under smooth transmission. Although the MAMF’s algorithm speed has been improved, the image is over-superimposed in the details. It results in serious detail loss and excessive saturation due to the addition of layers.
- The HRP uses nonlinear compression to optimize transmission and improve accuracy. At the same time, it uses logarithmic compression to simulate DCI of haze-free image and receive a polishing transmission. However, there is a lot of noise in the sky area, and a lot of noise is revealed after processing, resulting in halo phenomenon.
- Although the PDE proposes the method based on finding dark pixels, which effectively improved the overall brightness problem of the image and enriched the image details. But it has not fundamentally gotten rid of the constraints of the DCP. The image exhibit excessive saturation, darkened brightness, and details that are lost in several parts.
- The JCE combines three basic preprocessing techniques to create intermediate images to enhance contrast and use adaptability to handle complex and changing environments. In the fusion stage, it uses an adaptive kernel size based on fast structural block decomposition to fuse images processed by three basic preprocessing techniques. The method greatly improves the calculation time, but in terms of image quality. The three techniques are all based on image enhancement preprocessing techniques, which do not take into account the fact that fog exists and enhance the contrast of the image. Although the contrast of the effective information of the image is enhanced, the image quality is not effectively improved. Due to the fusion of multiple images, the details of the image are repeatedly superimposed, which causes the details to deepen, becomes blurred and is diﬃcult to observe. Secondly, the processing of the image causes the image saturation to increase, the depth of field to deepen, and chromatic aberration to occur in some details of the image. They results in poor image quality.
- The RSA optimizes the atmospheric illumination based on the Rayleigh Scatter theory. It enriches the effective information in the image, greatly improves the image quality, ensures the image details, and optimizes the color problem between the defogging image and the original foggy image. As the algorithm complexity increases, the time complexity of ours is relatively high.

In contrast, we optimize transmission in bright regions, which significantly improves the visual quality of the sky area. It closely resembles the original image in terms of color, which avoids color distortion and detail loss. To demonstrate the algorithm’s effectiveness further, we provide a comparison of five objective parameters: PSNR, SSIM, AVE, UQI, HCC and MS-SSIM.

The order in the table corresponds to the order in the figure. In the Table 2, the six objective parameters were introduced previously. By observing Table 2, we can see that the objective parameters measured by our method is higher than those of other methods. The reasons for this phenomenon are analyzed as follows. We can see from Fig 6 that the brightness of the image processed by methods other than the RSA is darker, and the DCP, MAMF, PDE and JCE process color loss in details. As a result, the outer frame of some areas appears black, which indicates that the atmospheric illuminations selected by these algorithms deviates from the actual atmospheric illuminations. It causes the transmission estimated by using inaccurate atmospheric illuminations to also deviate and images of depth of field estimation errors. These parameters result in inaccurate color restoration, loss of details, and deviations from the original image. The loss of details causes the objective parameters of PSNR, SSIM and UQI to decrease. Due to the inaccuracy of transmission, halos appear in the sky and a large amount of noise is introduced, which greatly affects the evaluation of parameters. We propose a method for modifying atmospheric illuminations and transmission, which greatly improves the above problems, restores images, enriches image details, and improves image quality.

**Table 2:** Objective evaluation parameters.

Objective parameters	Figure	DCP [12]	MAMF [30]	TDD [37]	PDE [38]	JCE [39]	RSA
PSNR	Town	63.3504	65.0244	63.9385	65.2371	60.2194	69.1043
	Mountain	61.9267	64.5344	65.4546	63.9213	62.4864	68.3010
	Snow mountain	60.9663	67.0110	63.9949	67.2597	62.9520	69.8626
	Forest	62.1010	65.3265	67.3813	63.6416	62.6516	70.3602
	City	60.0309	65.4584	67.6920	63.9520	60.2866	69.6575
	Pampkin	64.1027	65.6477	64.2120	63.6034	61.7083	69.1640
SSIM	Town	0.7513	0.7152	0.8524	0.8180	0.5189	0.9002
	Mountain	0.7408	0.6847	0.8466	0.7861	0.6883	0.8665
	Snow mountain	0.4888	0.6084	0.7229	0.7683	0.6428	0.8911
	Forest	0.7673	0.6897	0.9010	0.8072	0.6731	0.9260
	City	0.6411	0.7200	0.8592	0.7978	0.6546	0.9260
	Pampkin	0.8002	0.7089	0.8079	0.7772	0.6775	0.8848
AVE	Town	0.2761	0.3411	0.3319	0.3225	0.1915	0.3863
	Mountain	0.2781	0.3484	0.3620	0.3082	0.2724	0.3981
	Snow mountain	0.2703	0.3785	0.3897	0.3313	0.2950	0.4037
	Forest	0.2538	0.3400	0.3695	0.2826	0.2574	0.3892
	City	0.2108	0.3630	0.4029	0.2849	0.2970	0.4282
	Pampkin	0.2876	0.3424	0.3182	0.2800	0.2534	0.3864
UQI	Town	0.6624	0.7759	0.8483	0.7412	0.3475	0.8702
	Mountain	0.6885	0.8137	0.8510	0.7800	0.6989	0.9030
	Snow mountain	0.4963	0.7780	0.8389	0.7607	0.7069	0.8498
	Forest	0.7368	0.8619	0.9207	0.7819	0.7174	0.9508
	City	0.5206	0.7974	0.9288	0.7456	0.5502	0.9396
	Pampkin	0.7835	0.8483	0.8524	0.6814	0.6670	0.9217
HCC	Town	− 0 . 0471	0.0899	0.4502	0.0707	− 0 . 1372	0.5680
	Mountain	0.1487	0.1455	0.2441	0.2394	0.0388	0.5896
	Snow mountain	− 0 . 1143	0.2510	0.1984	− 0 . 0072	0.0481	0.5399
	Forest	0.0324	0.2536	0.5893	0.2048	0.0222	0.6633
	City	− 0 . 1470	0.0402	0.4256	0.1011	− 0 . 0637	0.5587
	Pampkin	0.1476	0.2578	0.4423	0.0294	-0.0159	0.5848
MS-SSIM	Town	0.8279	0.9076	0.8705	0.9140	0.6960	0.9669
	Mountain	0.8567	0.8916	0.8967	0.8672	0.8249	0.9559
	Snow mountain	0.8569	0.9491	0.9027	0.9437	0.6457	0.9744
	Forest	0.8582	0.8983	0.9241	0.8315	0.7909	0.9499
	City	0.8476	0.8795	0.8879	0.8802	0.7924	0.9009
	Pampkin	0.8268	0.9042	0.8475	0.8615	0.7763	0.9529

**Fig 7:**
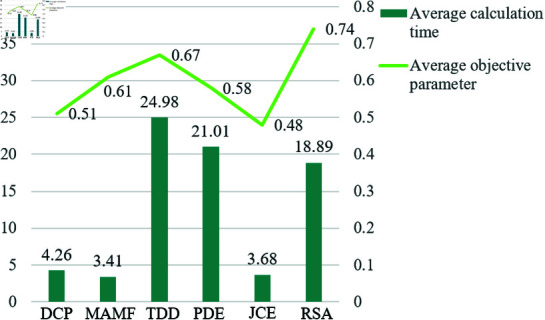
Performance comparison chart of each method. The bar chart represents the average running time of each method on some data, and the line chart represents the mean of the objective parameters of each method.

In order to conduct objective and multi-angle comparative experiments. We selects some images from the data set for time testing and calculates their average values. As shown in Fig 7.

By comparison, it can be found that the running time of DCP, MAMF and JCE methods is shorter, but the image quality restored by these three methods is relatively low, the color is generally biased, and the brightness and depth of field are too high, making it diﬃcult to observe the details.

The running time of TDD, PDE and ours is longer, but the image quality restored by these three methods is relatively high. Among them, ours has a shorter average running time and a higher quality of restored images, which is more advantageous.

From the above comparative experiments, it can be seen that the quality of the image is proportional to the time complexity of the method. Reducing the complexity of method while improving the image quality is a topic for future research in this direction.

## Conclusion

We introduce a color compensation dehazing method based on Rayleigh Scattering. The reasons for the low brightness, large color difference, unclear details, halo and other problems of the image processed by the traditional improved methods are analyzed. By analyzing BCP and DCP theory, the reasons for the inaccurate estimation of atmospheric illumination and transmission are summarized. According to BCP and DCP theory, RSA finds the areas with low-light image and optimizes restoration quality. On the one hand, according to Rayleigh scattering, when atmospheric light passes through the atmosphere, it will scatter due to the short wavelength of blue light, and its ability will be damaged to a large extent, which results in the difference between the color of the object and its true color. Since the transmission is estimated based on the atmospheric illumination, the deviation of atmospheric illumination will also cause errors in the transmission of the image. On the other hand, DCP fails in high-brightness areas, which results in inaccurate transmission estimation, unclear expression of processed image details, and aperture phenomenon around individual objects. By optimizing atmospheric illumination and transmission, we reduce the impact of the above problems on the image and improve the overall image quality. However, it should be noted that this method is not necessarily suitable for all haze images, which is also a direction for our future research. And RSA will take a little longer to process when encountering too large images. It is significantly important for our future research to optimize the method complexity, improve the method processing speed and robustness.
